# Are Adverse Childhood Experiences Associated With Being in the System of Care?

**DOI:** 10.3389/fpsyg.2022.909737

**Published:** 2022-06-24

**Authors:** Miriama Lackova Rebicova, Zuzana Dankulincova Veselska, Andrea Madarasova Geckova, Danielle E. M. C. Jansen, Jitse P. van Dijk, Sijmen A. Reijneveld

**Affiliations:** ^1^Department of Health Psychology and Research Methodology, Faculty of Medicine, PJ Safarik University, Kosice, Slovakia; ^2^Graduate School Kosice Institute for Society and Health, PJ Safarik University, Kosice, Slovakia; ^3^Department of Community and Occupational Health, University Medical Center Groningen, University of Groningen, Groningen, Netherlands; ^4^Institute of Applied Psychology, Faculty of Social and Economic Sciences, Comenius University in Bratislava, Bratislava, Slovakia; ^5^Theoretical Sociology - Department Sociology, Faculty of Behavioural and Social Sciences, University Medical Center Groningen, University of Groningen, Groningen, Netherlands; ^6^Olomouc University Social Health Institute, Palacky University in Olomouc, Olomouc, Czechia

**Keywords:** adverse childhood experiences, parent-related ACE, use of psychosocial care, adolescence, mental health

## Abstract

**Background:**

Adverse childhood experiences (ACEs) can cause serious mental problems in adolescents and therefore may expected to be associated with higher use of psychosocial care, potentially varying by type of specific ACE. The aim of our study is to explore the association of the number of ACE and types of specific ACE with entering and using psychosocial care.

**Methods:**

We used data from the Slovak Care4Youth cohort study, comprising 509 adolescents from 10 to 16 years old (mean age 13.2 years, 48.6% boys). We used logistic regression models adjusted for age, gender, and family affluence to explore the associations of number and type of specific ACE with the use of psychosocial care.

**Results:**

Having three or more ACE as well as experiencing some specific ACE (death of a mother/father, death of somebody else you love, problems of a parent with alcohol or drugs, conflicts or physical fights between parents, and separation/divorce of parents) increased the likelihood of using psychosocial care. Regarding experience with the death of somebody else you love, we found a decreased likelihood of the use of psychosocial care.

**Conclusion:**

Experiencing ACE above a certain threshold (three or more) and parent-related ACE increase the likelihood of adolescent care use.

## Introduction

Adverse childhood experiences (ACEs) represent various negative events during a child’s development (DeLisi et al., 2017; Sheikh, 2018; Lackova Rebicova et al., 2019, 2020; Paclikova et al., 2019). Accumulation of ACEs has been shown to have long-lasting effects on adolescents’ mental health (Danese and McEwen, 2012; Lackova Rebicova et al., 2020), which may be associated with their enrolment into the system of care (Paclikova et al., 2020) and consequent utilization of the system of care (Aalsma et al., 2016). As a dose–response was found between ACE and adolescents’ mental health (Lackova Rebicova et al., 2020), we would expect a similar pattern when it comes to use of psychosocial care. However, currently evidence is lacking on the influence of the number of ACE on being in and using psychosocial care among adolescents.

ACE regard different types of negative events with different levels of severity such as abuse and/or neglect of a child, domestic violence toward a youth’s mother, household substance abuse, household mental illness, parental separation/divorce, and other events. They represent harm that affects the child either directly (such experiences may include neglect and abuse) or indirectly by affecting the environment in which they live (such as family mental disorders, parental divorce, family psychoactive substance use, etc.; Danese and McEwen, 2012; DeLisi et al., 2017; Sheikh, 2018; Paclikova et al., 2019). It has been suggested that ACE can be divided into several groups with differing impacts—ACE related to parents, to other family members and to other negative events—and those types of ACE that are directly related to parents (Finkelhor et al., 2013; Schwartz et al., 2019; Wright and Schwartz, 2021) seem to have a more significant impact on a child’s life (Finkelhor et al., 2013; Schwartz et al., 2019; Wright and Schwartz, 2021). Recent studies have revealed that in particular conflicts between parents, divorce, or parents drinking alcohol seem to have a negative influence on adolescents’ mental health (Maier and Lachman, 2000; Bevilacqua et al., 2021). Such parent-related ACE may have a stronger impact, as parents might be more self-centered and as a result less sensitive and responsive to their children. Therefore, family functioning is being compromised and other sources for dealing with the problems are needed (Morris et al., 2007).

The theoretical background of such findings might be in the Complex trauma theory. Complex trauma in children or adolescents is a result of exposure to severe adverse experiences, often in the family system—from persons who should be sources of security, protection and stability, with the consequence in the form of disruption of the sense of self leading to mental health problems and the need for psychosocial care (Cook et al., 2017). As regards other types of ACE, a lot of adolescents have experienced the death of somebody else he or she loves, which can have a significant impact as well (Johnson et al., 2017; Pachalla et al., 2020). Overall, ACE have been shown to have an impact (Lackova Rebicova et al., 2020), but it may be expected that the influence of a specific type of ACE is different (Negriff, 2020; Mackova et al., 2021). Studies that comprehensively address the association of specific types of ACE on the use of psychosocial care are lacking. To sort out the effect of other related ACE requires an assessment per ACE adjusted for the associations of the other ACE.

To summarize, the previous studies have mostly explored the association of ACE with adolescent mental health and/or enrolment in care for mental health problems in general (Lackova Rebicova et al., 2019; LaBrenz et al., 2020; Paclikova et al., 2020; Meeker et al., 2021). Evidence is lacking on the potential dose–response association between ACE and the use of psychosocial care. The same holds for understanding which specific ACE are most strongly associated with use of psychosocial care when adjusted for each other. Therefore, the aim of our study was to explore the association between ACE (number and type of specific ACE) and entering and using the system of psychosocial care.

## Materials and Methods

### Sample and Procedure

We used data from the Slovak Care4Youth cohort study that comprises both adolescents from the general community and adolescents entering psychosocial care, i.e., two subsamples. The respondents had to meet the following criteria: adolescents should be aged 10–16 years old, should be able to understand the Slovak language, should be able to fill out the questionnaires on their own, and should come from the Kosice region in eastern Slovakia.

**Figure 1:**
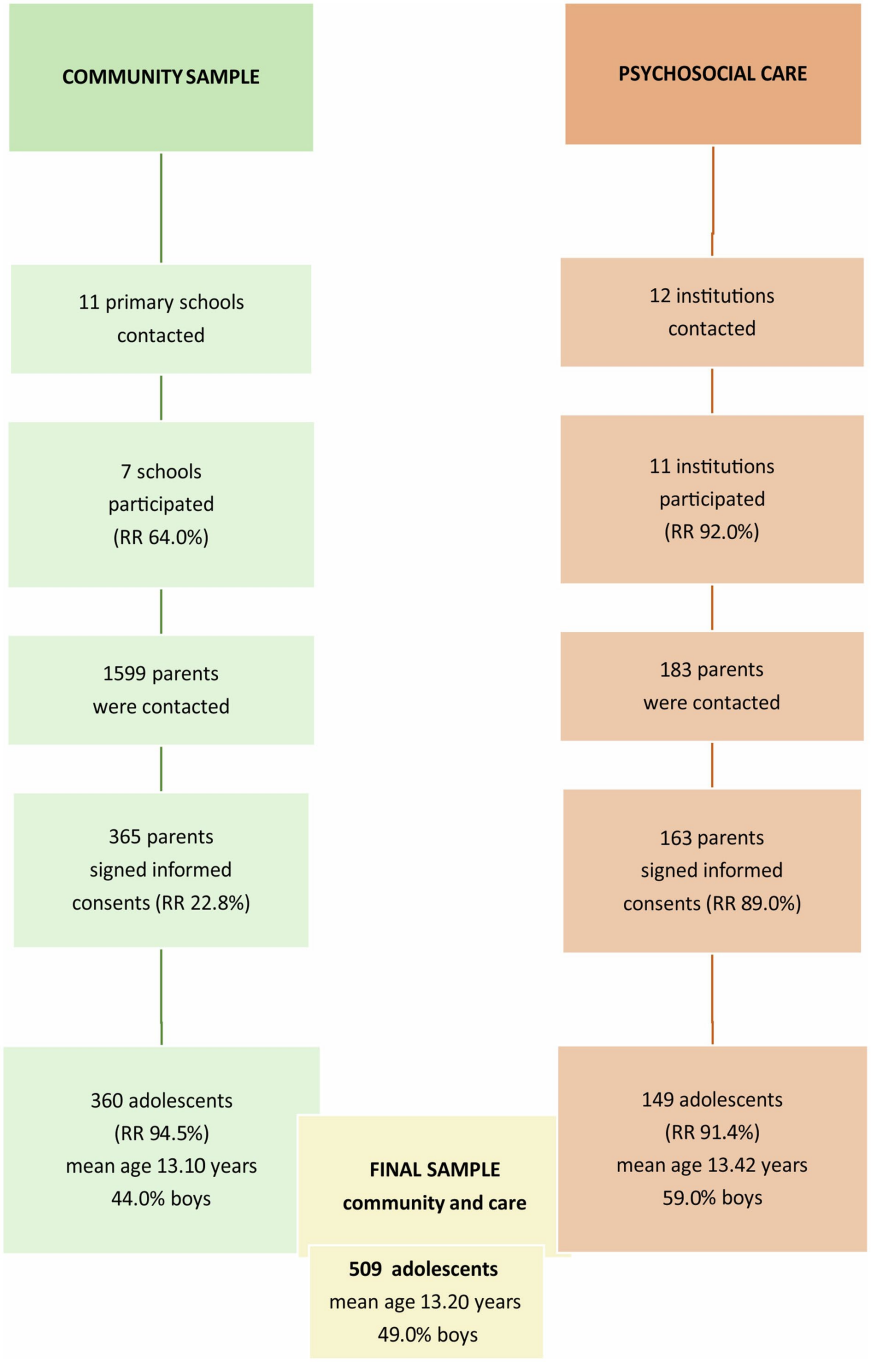
Flowchart outlining the process of recruitment of participants from the Care4Youth study, Slovakia 2017, 10–16 years old, *N* = 509.

Participants (10–16 years old) from the community were recruited *via* randomly chosen primary schools in the Kosice region in eastern Slovakia; they were approached from January until June 2017 *via* a two-stage sampling. In the first stage, we contacted the schools; in the second stage, the parents or legal representatives of the pupils were contacted. After being thoroughly informed about the study and participation in the study, parents (when children were <18, we had to ask parents only) were asked to provide us with signed informed consent on behalf of their children. Detailed information on response rates and the number of participants with basic descriptive information is shown in Figure 1.

Participants from the care system were recruited *via* institutions providing psychosocial care for adolescents with emotional and behavioral problems in the Kosice region in eastern Slovakia. These were approached from January 2017 until December 2018 using a two-step sampling. In the first step, we contacted institutions and, in the second step, we contacted the parents or legal representatives of the adolescents. Parents received full information on the study and provided a signed informed consent on behalf of their children. Detailed information on participants’ flow with basic descriptive information is shown in Figure 1.

For the purpose of this study, we used a final sample consisting of 509 adolescents (mean age 13.20 years, 48.6% boys). Respondents with missing responses on the variables to be studied were excluded. The study was approved by the Ethics Committee of the Medical Faculty at the Pavel Jozef Safarik University in Kosice (protocol 2N/2015).

### Measures

*Adverse childhood experiences* (*ACE*s) were measured by a series of questions on events: “Have you ever experienced any of the following serious events?” (Death of a brother/sister, Death of your father/mother, Death of somebody else you love, Long or serious illness of yourself, Long or serious illness of one of your parents or of someone else close to you, Problems of one of your parents with alcohol or drugs, Repeated serious conflicts or physical fights between your parents, Separation/divorce of your parents, and Separation of your parents due work abroad). These questions were derived from the ISRD 2: Standard Student Questionnaire and piloted in the Slovak context (ISRD2 Working Group, 2005; Lackova Rebicova et al., 2020). The response categories were “Yes” and “No.” We created a sum score for the number of ACE experienced, with a higher score indicating more ACE. Consequently, we categorized the number of ACE into three categories: no ACE, one or two ACE and three or more ACE. We used in our analysis the cumulative ACE (three categories: 0 ACE vs. 1–2 ACE vs. 3 and more ACE) and also the specific ACE, as listed above (two categories: No/Yes).

*Psychosocial care use* regarded as being either in the care sample or in the community sample and using psychosocial care from the institutions providing social and mental health care (e.g., counseling centers, in-patient and out-patient psychiatric care, out-patient psychological care, and state and non-profit organizations providing social services) according to information provided by the parents over the past 12 months.

We further assessed age, gender, and socioeconomic position as potential confounders. *Socioeconomic position* (*SEP*; Inchley et al., 2018) was used as a measure of socioeconomic status and was measured by a previously validated tool (Ekehammar et al., 1987) on a 10-point scale (0—the worst, 10—the best); the adolescents were asked to assess where they see their families on this ladder according to their financial status (Adler et al., 2000). To illustrate what is meant, a description was provided, e.g., about how well-off their family is, how much money the family had, what level of education their parents had achieved and how profitable the work of their parents is.

### Statistical Analyses

First, we described the sample using descriptive statistics. Second, we assessed the crude association of the number and type of the specific ACE with the use of psychosocial care (Model 1). Third, we assessed the univariate association of the number and type of specific ACE with use of psychosocial care adjusted for gender, age and SEP (Model 2). Finally, we assessed the association of type of specific ACE with use of psychosocial care adjusted for gender, age and SEP (Model 3). For the last three steps, we used logistic regression models. Statistical analyses were performed using SPSS v.25.

## Results

### Background Characteristics

The background characteristics of the sample are presented in Table 1. Our full sample consisted of 509 adolescents aged 10–16 years old (boys: 48.6%; *n* = 240). Of these, 360 were in the community sample (boys: 44.1%) and 149 in the care sample (boys: 59.1%).

**Table 1:** Descriptive statistics of the sample (Care4Youth study, Slovakia 2017, 10–16 years old, *N* = 509).

	Total	Care sample	Community sample
	*N* = 509	*N* = 149	*N* = 360
**Gender (*N*, %)**			
Boys	240 (48.6)	88 (59.1)	152 (44.1)
**Age (mean, SD)**	13.19 (1.55)	13.42 (1.77)	13.10 (1.44)
**Socioeconomic position (mean, SD)**	6.80 (1.80)	5.97 (2.09)	7.15 (1.55)
**Number of ACE (*N*, %)**			
0 ACE	140 (28.9)	34 (23.1)	106 (31.5)
1–2 ACE	254 (52.5)	70 (47.6)	184 (54.6)
3 or more ACE	90 (18.6)	43 (29.3)	47 (13.9)
**Type of specific ACE (*N*, %)**			
Death of a brother/sister (yes)	8 (1.7)	3 (2)	5 (1.5)
Death of a mother/father (yes)	33 (6.8)	23 (15.6)	10 (3)
Death of somebody else you love (yes)	203 (41.9)	50 (34)	153 (45.4)
Long or serious illness of yourself (yes)	45 (9.3)	16 (10.9)	29 (8.6)
Long or serious illness of a parent or of someone else close to you (yes)	142 (29.3)	35 (23.8)	107 (31.8)
Problems of a parent with alcohol or drugs (yes)	52 (10.7)	30 (20.4)	22 (6.5)
Repeated serious conflicts or physical fights between your parents (yes)	72 (14.9)	37 (25.2)	35 (10.4)
Separation/divorce of your parents (yes)	110 (22.7)	52 (35.4)	58 (17.2)
Separation of your parents due work abroad (yes)	35 (7.2)	17 (11.6)	18 (5.3)
**Use of psychosocial care**	186 (36.5)	149 (100.0)	37 (10.3)

N - number of respondents; ACE - adverse childhood experiences; and SD - standard deviation.

### Association of Use of Psychosocial Care With the Number of ACE and With the Type of ACE

We assessed the crude association of number of ACE and type of ACE with the use of psychosocial care using logistic regression models (Table 2, Model 1). Having three or more ACE as well as experiencing some of type of specific ACE (Death of a mother/father; Death of somebody else you love, Problems of one of your parents with alcohol or drugs; conflicts or physical fights between parents; Separation/divorce of parents) increased the likelihood of use of psychosocial care (OR = 8.51; 0.54; 3.59; 3.31; and 2.22, respectively). In comparison, the ACE Death of somebody else you love decreased the likelihood of use of psychosocial care (OR = 0.54).

**Table 2:** Association between ACE (number and type of specific) and use of psychosocial care; results from logistic regression models leading to odds ratios (OR) and 95% confidence intervals (95% CI), crude (Model 1), adjusted for confounders (age, gender and socioeconomic position; Model 2), and adjusted for the associations with other ACE (Model 3; Care4Youth study, collected in 2017–2018, Slovakia, 10–16 years old, *N* = 509).

	Model 1	Model 2	Model 3
	OR (95% CI)	OR (95% CI)	OR (95% CI)
**Number of ACE**			
0	Ref.	Ref.	
1–2	1.22 (0.79|1.89)	0.97 (0.61|1.55)	
3 or more	2.49 (1.44|4.31)^***^	1.80 (1.00|3.23)^*^	
**Type of specific ACE**			
Death of a brother/sister (vs. no)	0.99 (0.23|4.18)	0.68 (0.14|3.37)	0.47 (0.08|2.77)
Death of a mother/father (vs. no)	8.51(3.44|21.05)^***^	11.84 (4.21|33.30)^***^	11.37 (3.80|33.66)^***^
Death of somebody else you love (vs. no)	0.54 (0.37|0.79)^***^	0.49 (0.32|0.73)^***^	0.50 (0.32|0.80)^**^
Long or serious illness of yourself (vs. no)	1.23 (0.66|2.28)	1.03 (0.53|1.99)	1.20 (0.59|2.43)
Long or serious illness of a parent or of someone else (vs. no)	0.75 (0.50|1.13)	0.65 (0.42|1.01)	0.71 (0.43|1.19)
Problems of a parent with alcohol or drugs (vs. no)	3.59 (1.96|6.57)^***^	2.71 (1.42|5.14)^***^	1.19 (0.53|2.70)
Repeated serious conflicts or physical fights between your parents (vs. no)	3.31 (1.97|5.56)^***^	2.71 (1.55|4.74)^***^	2.20 (1.10|4.37)^*^
Separation/divorce of your parents (vs. no)	2.22 (1.44|3.42)^***^	1.94 (1.24|3.06)^***^	1.68 (1.00|2.84)^*^
Separation of your parents due work abroad (vs. no)	1.82 (0.91|3.63)	1.27 (0.67|2.65)	1.00 (0.44|2.24)

* *p* < 0.05;

** *p* < 0.01;

*** *p* < 0.001.

### Association of Use of Psychosocial Care With the Number of ACE and With the Type of ACE Adjusted for Gender, Age, and SEP

Model 2 in Table 2 shows that having three or more ACE (OR = 1.80) as well as experiencing some type of specific ACE (Death of a mother/father, Problems of one of your parents with alcohol or drugs, Conflicts or physical fights between parents, and Separation/divorce of parents) increased the likelihood of use of psychosocial care (OR = 11.84; 2.71; 2.71; and 1.94, respectively). In contrast, Death of somebody else you love decreased the likelihood of use of psychosocial care (OR = 0.49).

### Association of Use of Psychosocial Care With Type of ACE After Adjustment for Gender, Age, and SEP

Finally, Model 3 in Table 2 shows that Death of a mother/father, Conflicts or physical fights between parents; Separation/divorce of parents increased the likelihood of use of psychosocial care (OR = 11.37; 2.20; and 1.68, respectively). Experience with Death of somebody else you love still reduced the likelihood of use of psychosocial care (OR = 0.50).

## Discussion

We found that a higher number of ACE (three or more) increased the likelihood of using psychosocial care among adolescents. For specific ACE, the parent-related ones increased the likelihood of using care in particular. One specific ACE—Death of somebody else you love—decreased this likelihood.

We found that the use of psychosocial care by adolescents was more likely when experiencing an accumulation of ACE (three or more) than when experiencing no, one or two ACE. This is in line with recent research, showing an accumulation of ACE to have a deleterious effect on the mental health of adolescents (Bevilacqua et al., 2021; Meeker et al., 2021). Previously, we reported a similar dose–response association between ACE and emotional and behavioral problems (Lackova Rebicova et al., 2020). The occurrence of mental problems and problems related to ACE directly can lead to a need for psychosocial care among adolescents (Nanninga, 2018). The higher use of psychosocial care is likely explained by this occurrence of problems, with the sources in family/school/community being no longer sufficient to manage it. Adolescents having experienced ACE and few resources are more likely to enter and use psychosocial care (Nanninga, 2018), including specific care from professionals (Negriff, 2020; Mackova et al., 2021).

We further found a different effect of certain specific ACE on adolescents’ use of psychosocial care, with care use being more likely for only three specific parent-related ACE (Death of a mother/father; Conflicts or physical fights between parents; and Separation/divorce of parents). These findings are in line with existing, though partial, knowledge on the association between such specific type of ACE and worsening mental health leading to use of psychosocial care (Murphy et al., 2016; LaBrenz et al., 2020). As is stated in the Tripartite model of the impact of the family on children’s emotion regulation and adjustment, the ability for emotional regulation and subsequent positive adjustment and development in mental health among adolescents is influenced *via* the parent–child attachment relationship, parenting style and the marital relationship (Morris et al., 2007). Once the parent-related ACE occurs, threating the attachment relationship, parenting style and marital relationship, the family’s potential for emotional regulation and adjustment declines, and the adolescent might need additional help from professionals in the system of care. Thus, our findings show the important role of ACE related to parents in association with use of psychosocial care and is in contrast with our finding on the ACE – Death of a mother/father where loss of parents as a crucial attachment figures might activate further discomfort and disorganization in adolescents, which in turn, might increase the use of psychosocial care.

To our surprise, we also found that the death of somebody else you love decreased the likelihood of being in and using the system of care, both crude and fully adjusted. Our findings are in contrast with previous research, which typically have described the association of loss of a significant other due to death with negative outcomes (Johnson et al., 2017). An interpretation might regard the process during which the death of a significant other encourages greater family, peer, school, and/or community activation in relation to a child. As a result, a child may receive additional support and attention and such a situation may result in a positive adjustment and personal growth, making the utilization of the system of care less likely (Pachalla et al., 2020). This is consistent with our findings that a specific ACE—Death of somebody else you love—decreased the use of psychosocial care among adolescents.

### Strengths and Limitations

The main strength is that our study involved adolescents from both the community and the system of care and covered a range of ACE. However, our study has some limitations too. A first limitation regards the cross-sectional design of this study; this makes it impossible to formulate conclusive statements about causality. A second limitation may regard our use of self-reported data for measuring ACE and SEP. However, the previous studies have shown the validity of self-reported measurement of ACE (Meinck et al., 2017; Lackova Rebicova et al., 2019, 2020) and SEP (Ekehammar et al., 1987; Adler et al., 2000).

### Implications

Our study showed that the number of ACE is associated with the using of care among adolescents, with more ACE having a stronger association with use of psychosocial care, and that parent-related ACE increase the likelihood of being in and using the system of care among adolescents in particular. These results imply a need to help adolescents with cumulative ACE and with parent-related ACE by strengthening support in the form of external resources (extended family, community, and school). Moreover, to address above mentioned limitations of our study, we suggest to use in the future research a longitudinal follow-up design and the triangulation of data sources with data from parents, teachers or care providers being collected and analyzed.

### Conclusion

Accumulation of ACE above a certain threshold (three or more) and specific, parent-related, ACE increase the likelihood of adolescent using the system of care. Special attention should be given to those adolescents, as they could be considered as an at-risk population.

## Data Availability Statement

The raw data supporting the conclusions of this article will be made available by the authors, without undue reservation.

## Ethics Statement

All procedures performed in the study were in accordance with the 1964 Declaration of Helsinki and its later amendments or comparable ethical standards. The study was approved by the Ethics Committee of the Medical Faculty at the PJ Safarik University in Kosice (protocol 16/2017). Written informed consent to participate in this study was provided by the participants’ legal guardian/next of kin.

## Author Contributions

MR participated in the design of the study and coordination, drafted the manuscript, analyses, and interpretation of the data. ZV participated in the design and coordination of the study and interpretation of the data, helped to draft the manuscript, and provided supervision. SR, AG, DJ, and JD participated in the interpretation of the data, contributed with their comments to the final version, and provided supervision. All authors contributed to the article and approved the submitted version.

## Funding

This work was supported by the Research and Development Support Agency under contract nos. APVV-15-0012 and APVV-21-0079 and the Scientific Grant Agency of the Ministry of Education, Science, Research and Sport of the Slovak Republic and the Slovak Academy of Sciences, VEGA reg. no. 1/0177/20.

## Conflict of Interest

The handling editor declared a shared affiliation with the authors [AMG & JVD] at the time of the review.

The authors declare that the research was conducted in the absence of any commercial or financial relationships that could be construed as a potential conflict of interest.

## Publisher’s Note

All claims expressed in this article are solely those of the authors and do not necessarily represent those of their affiliated organizations, or those of the publisher, the editors and the reviewers. Any product that may be evaluated in this article, or claim that may be made by its manufacturer, is not guaranteed or endorsed by the publisher.
